# Exploring pangenomic diversity and CRISPR-Cas evasion potential in jumbo phages: a comparative genomics study

**DOI:** 10.1128/spectrum.04200-23

**Published:** 2024-09-12

**Authors:** Sharayu Magar, Vaishnavi Kolte, Gaurav Sharma, Sutharsan Govindarajan

**Affiliations:** 1 Department of Biological Sciences, SRM University AP, Amaravati, Andhra Pradesh, India; 2 Institute of Bioinformatics and Applied Biotechnology (IBAB), Bengaluru, Karnataka, India; 3 Department of Biotechnology, Indian Institute of Technology Hyderabad, Hyderabad, India; The Hebrew University of Jerusalem, Rehovot, Israel

**Keywords:** jumbo phage, phage diversity, pan-genome, phylogeny, genomics, CRISPR-Cas

## Abstract

Jumbo phages are characterized by their remarkably large-sized genome and unique life cycles. Jumbo phages belonging to Chimalliviridae family protect the replicating phage DNA from host immune systems like CRISPR-Cas and restriction-modification system through a phage nucleus structure. Several recent studies have provided new insights into jumbo phage infection biology, but the pan-genome diversity of jumbo phages and their relationship with CRISPR-Cas targeting beyond Chimalliviridae are not well understood. In this study, we used pan-genome analysis to identify orthologous gene families shared among 331 jumbo phages with complete genomes. We show that jumbo phages lack a universally conserved set of core genes but identified seven “soft-core genes” conserved in over 50% of these phages. These genes primarily govern DNA-related activities, such as replication, repair, or nucleotide synthesis. Jumbo phages exhibit a wide array of accessory and unique genes, underscoring their genetic diversity. Phylogenetic analyses of the soft-core genes revealed frequent horizontal gene transfer events between jumbo phages, non-jumbo phages, and occasionally even giant eukaryotic viruses, indicating a polyphyletic evolutionary nature. We categorized jumbo phages into 11 major viral clusters (VCs) spanning 130 sub-clusters, with the majority being multi-genus jumbo phage clusters. Moreover, through the analysis of hallmark genes related to CRISPR-Cas targeting, we predict that many jumbo phages can evade host immune systems using both known and yet-to-be-identified mechanisms. In summary, our study enhances our understanding of jumbo phages, shedding light on their pan-genome diversity and remarkable genome protection capabilities.

**IMPORTANCE:**

Jumbo phages are large bacterial viruses known for more than 50 years. However, only in recent years, a significant number of complete genome sequences of jumbo phages have become available. In this study, we employed comparative genomic approaches to investigate the genomic diversity and genome protection capabilities of the 331 jumbo phages. Our findings revealed that jumbo phages exhibit high genetic diversity, with only a few genes being relatively conserved across jumbo phages. Interestingly, our data suggest that jumbo phages employ yet-to-be-identified strategies to protect their DNA from the host immune system, such as CRISPR-Cas.

## INTRODUCTION

Bacteriophages, which are viruses that infect bacteria, are considered the most abundant biological entities on Earth. Nearly 96% of bacteriophages contain dsDNA as their genetic material and their size ranges from 14.2 kb to 642.4 kb (1, 2). Less than 1% of phages have large genomes exceeding 200 kb in size. Such phages are commonly referred to as jumbo phages because of their unusually large genome and capsid size (3, 4). A recent study suggested that phages with genome size >180 kb can also be potentially considered jumbo phages due to their shared proteome with phages having genome size >200 kb (5). Because of their unique biological characteristics and therapeutic potential, jumbo phages have attracted considerable attention in recent years. This has led to the isolation of hundreds of novel jumbo phages and the development of methods for their isolation (6–10) Metagenomics-based analyses support the widespread distribution of jumbo phages in various environments, including the ocean, human microbiome, wastewater, and soil (11, 12).

Jumbo phages are strictly lytic and depict distinct structures, genetic content, genome organization, and infection mechanisms from other bacteriophages. Based on their morphology, they were earlier classified into either family Myoviruses or Siphoviruses, according to the older classification system (4, 5). Additionally, jumbo phages have been classified as ⏀KZ-like or T4-like phages based on their morphology and host range (13, 14), and genomic analysis has classified them into 11 clusters and five singletons (4). More recently, phylogenetic analysis has classified jumbo phages into three distinct groups based on their replication mechanisms (5). In some cases, the virions of jumbo phages have unique morphological features, including tentacle-like head fibers from the capsid (1) and whisker-like structures along the tail (15). The exact functions of these unusual virion-associated appendages are not known, but they have been proposed to assist host attachment. In addition to external appendages, some jumbo phages contain unique components encapsulated within their capsids. For example, *Salmonella* jumbo-phage SPN3US contains “mottled-capsids,” which are filled with protein-like materials in addition to DNA (16). Phage ⏀KZ packs several proteins including a virion RNA polymerase and “inner body” proteins, which are released into the host during ejection. The “inner body” of ⏀KZ is a proteinaceous spool-like structure that wraps around the phage DNA inside the capsid. The inner body is conserved in a few jumbo phages, and they are suggested to be important for DNA organization, ejection, and protection, but their exact structure and function remain elusive (17, 18) Other features of jumbo phages include the presence of one or more tRNAs, DNA polymerases, metabolic genes, ribosomal genes, and novel genes involved in DNA protection (5).

A remarkable aspect of ⏀KZ-like jumbo phages is their ability to overcome DNA-targeting bacterial immune systems such as CRISPR-Cas and restriction modification (19, 20). During infection, ⏀KZ-like jumbo phages form an elaborate proteinaceous phage nucleus that shields phage DNA from the host nucleases. The phage nucleus is majorly composed of a single protein Gp54 (for ⏀KZ) named ChmA (for chimallin) (21, 22). Recent structural investigations have revealed that ChmA self-assembles to form a closed compartment made of quasi-symmetric tetramers with pores within their lattice structure. Pores have been suggested to mediate the selective transport of proteins and mRNAs across the shell (21, 22), and recent investigations have uncovered novel factors mediating protein and RNA transport within the phage nucleus (23–25). Most importantly, the micrometer-sized phage nucleus compartment is localized within the infected cell by a jumbo phage-encoded tubulin-like protein, PhuZ (26). ChmA and PhuZ together have been shown to orchestrate two most important functions within the infected cell namely phage genome protection and molecular transport, respectively (27, 28). A recent study categorized certain jumbo phages as Chimalliviridae, characterized by the presence of ChmA and a set of core genes in this viral family (29, 30). As ChmA and PhuZ are not widely conserved across all jumbo phages, it would be interesting to know if other jumbo phage proteins are capable of genome protection. Moreover, the nucleus-like phage shell is also suggested to act as a genetic barrier limiting inter-phage recombination (31). A recent genomic study highlighted the presence of various defense-related proteins within jumbo phage genomes, including DNA and RNA repair proteins, DNA modification factors such as methyltransferase, and the incorporation of uracil instead of thymine (5, 32) which might contribute to genome protection from nucleic acid-targeting host immune enzymes such as CRISPR-Cas.

The evolution of jumbo phages is less explored and more enigmatic. The current model suggests that jumbo phages evolved independently from smaller phages (4, 5). This is mainly due to the fact that jumbo phages are found in myoviruses and siphoviruses families, which evolved independently. Similarly, several core structural proteins, including capsid proteins, are conserved in jumbo phages, as well as in smaller phages (4). Analysis of conserved protein families and phylogenetic patterns also suggested multiple origins of jumbo phages from small progenitors (5). It has been proposed that genome expansion in jumbo phages occurs through the acquisition of host genes via horizontal gene transfer (33). Genome expansion by fusion of two smaller ancestral phage genomes has also been proposed (4, 34). However, the prevalence and extent to which such mechanisms contribute to the genome expansion of jumbo phages have not yet been studied in detail.

In this study, we explore the pan-genome and CRISPR-Cas evading potential of 331 jumbo bacteriophages having complete genomes. We show that jumbo phages exhibit extensive genetic heterogeneity in their genomes as revealed by the presence of soft core, accessory, and unique genes across them. Furthermore, our data suggest that several classes of jumbo phages, not related to Chimalliviridae, are also capable of evading the CRISPR-Cas system, possibly involving novel mechanisms.

## RESULTS

### General information on jumbo phages

A total of 331 jumbo phage genomes with genome size exceeding 180 kb were extracted from the NCBI Virus database (https://www.ncbi.nlm.nih.gov/labs/virus/vssi/#/) as of August 30, 2021, along with their taxonomic classification information (Table S1). Phages with a genome size range of 180 to 200 kb are considered potential jumbo phages (5), whereas phages with a genome size range of 200 to 500 kb and above 500 kb are categorized as strict jumbo phages and megaphages, respectively (11, 35). In our study, we considered all phages above 180 kb in size as jumbo phages in order to understand their genomic and evolutionary relatedness. These jumbo phages were categorized based on the most recent updates made by the ICTV in 2022 (36). Out of the 331 jumbo phages, 77 were classified into three families: Kyanoviridae (37/77), Straboviridae (38/77), and Eucampyvirinae (2/77), whereas the remaining 254 jumbo phages have not been assigned to a family yet. Among the 331 jumbo phages, 216 were further divided into 88 genera, whereas the remaining phages remained unclassified within the order Caudoviricetes.

The jumbo phage genomes vary in size, ranging from 180,111 bp (*Cyanophage* S-RIM14) to 551,627 bp (*Prevotella* phage Lak-B8), with a median size of 244,950 bp (average: 263,430 bp). These jumbo phages typically encode 326 proteins on the median scale (average: 358 proteins), with protein counts ranging from 216 (*Cyanophage* S-RIM14) to 911 (*Prevotella* phage Lak-B8). The GC content of these 331 jumbo phages ranged from 41.1% (*Cyanophage* S-RIM14) to 26% (*Prevotella* phage Lak-B8), with a median GC content of 43% (average: 43.6%).

### Functional annotation of proteins encoded by jumbo phages

**Fig 1:**
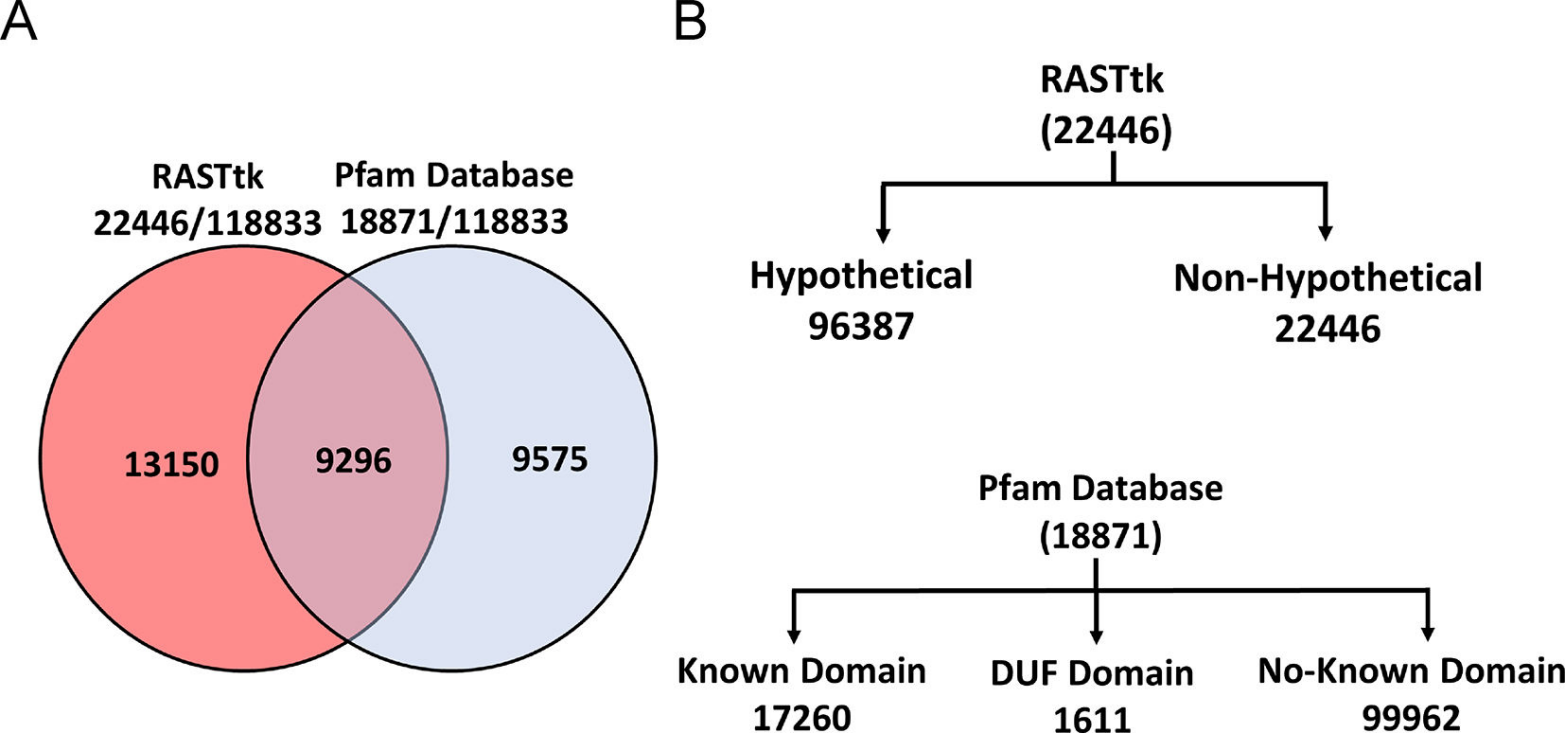
Functional annotation of proteins encoded by jumbo phages. (**A**) The Venn diagram illustrates the details of functional protein annotation within jumbo phages, conducted using two distinct methods: RASTtk and the Pfam database. (**B**) Further classification of these ORFs involves categorizing them into hypothetical and non-hypothetical using the RASTtk tool, whereas the Pfam database categorizes them into known domain, DUF-domain, and no-known domain.

Using RASTtk, we predicted 118,833 ORFs encoded by 331 jumbo phages. RASTtK and Pfam-mapping were employed to predict the functions of these proteins based on their sequence similarities and conserved domains (37, 38). In total, these two tools identified the potential functions of 32,021 ORFs (26.94%), with each algorithm yielding different numbers and percentages of overlaps (Fig. 1A and B). RASTtk annotated the highest number of proteins with known/predicted functions (22,446), followed by Pfam (18,871). We were able to attribute a putative function to ~18% of the RASTtk annotations proteins, whereas the remaining ORFs were hypothetical proteins with no known functions. The ORFs predicted by the Pfam Database were categorized into three groups: 14.5% of proteins with known domains (17,260), 1.3% of proteins with DUF domains (1,611), and 84.1% of proteins with no known domains (99,962). Based on our analysis using two different approaches (Table S2), we observed that nearly 73% of the jumbo phage genomes contain proteins of unknown function, representing phage dark matter.

### Pan-genome analysis and identification of soft-core genes

**Fig 2:**
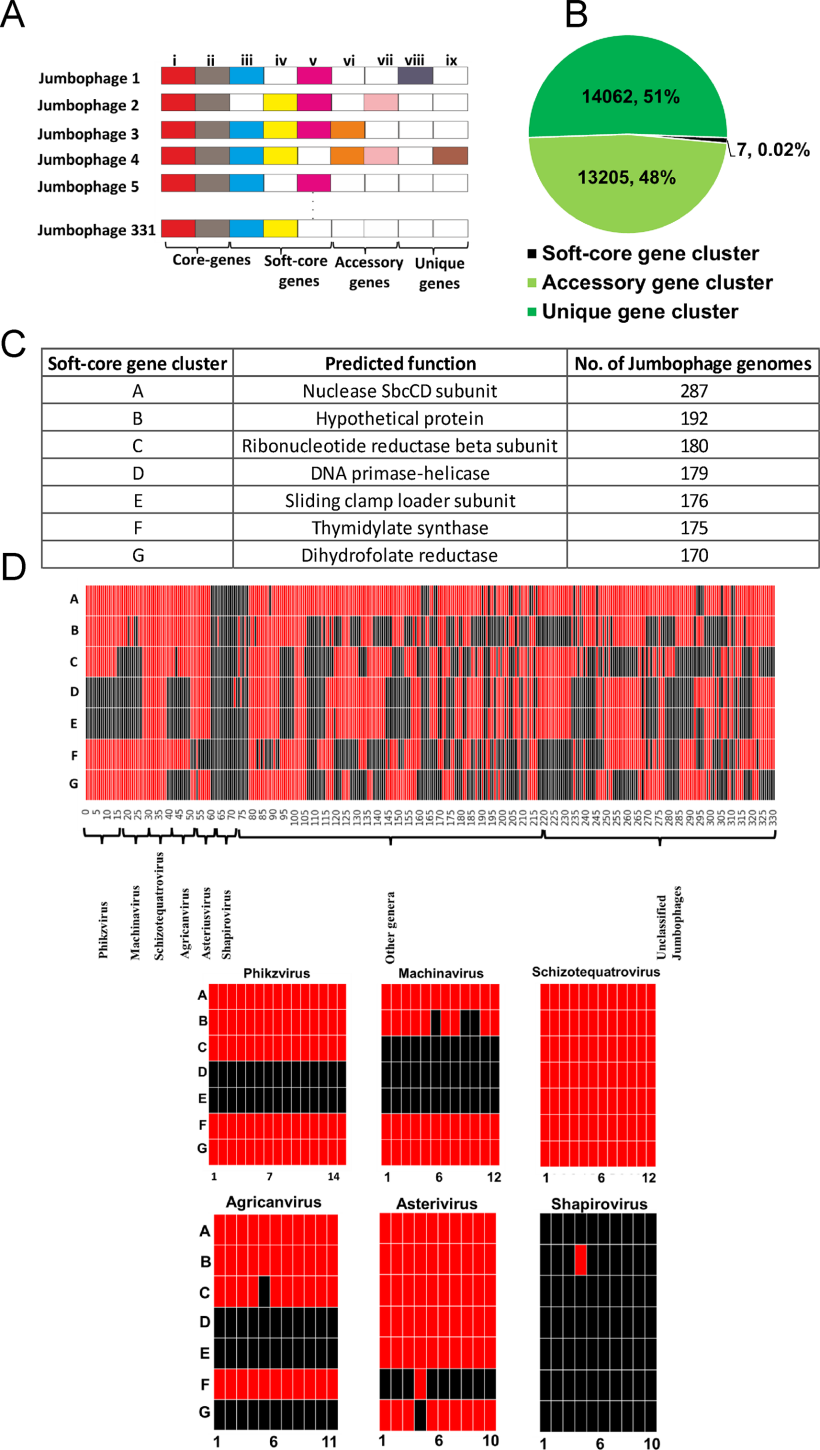
Pan-genome of jumbo phages. (**A**) Model depicting pan-genome distribution of jumbo phages. (**B**) Pan-genome summary statistics. Pie chart displays the numbers of total genes within each category with percentages. (**C**) The table provides details of genes classified within the soft-core gene category. It includes information about their predicted functions and indicates the number of jumbo phage genomes in which these genes are present. (**D**) The heatmap represents the distribution of soft-core genes across 331 jumbo phage genomes, denoted as A, B, C, D, E, F, and G. In the heatmap, the color red indicates the presence of a gene, whereas black signifies its absence (**D**). (**E**) illustrates the distribution of soft-core genes on jumbo phage genera that feature more than 10 phages. These prominent genera include *Phikzvirus*, *Machinavirus*, *Schizotequatrovirus*, *Agricavirus*, *Asterivirus*, and *Shapirovirus*.

The pan-genome encompasses all genes present within a particular group of organisms, which are divided into distinct gene clusters: core genes, conserved across all organisms studied; accessory genes, found in some members of the group; and unique genes, specific to individual species (39) (illustrated in Fig. 2A). In our study, pan-genome analysis was employed to identify orthologous gene pairs among the 331 jumbo phages using ProteinOrtho V.6.0 (40). Pan-genome analysis using 25% identity, 50% coverage, and 0.95 similarity parameters identified the highest number (27,275) shared orthologous gene clusters within the 331 jumbo phages.

This analysis could not discern any core genes universally conserved among all jumbo phages. It must be noted that prior studies have suggested the conservation of a few genes, such as the terminase large subunit and chromosomal end-processing complex SbcCD, across all jumbo phages (5); however, these studies considered functional conservation rather than sequence conservation. Nevertheless, our analysis revealed seven gene clusters conserved in more than 50% of jumbo phages, collectively representing 0.02% of the entire pan-genome (Fig. 2B). We categorize them as “soft-core” genes, given their relative conservation but non-universal presence among all jumbo phages. Notably, the chromosomal end-processing complex SbcCD D-subunit emerged as the most prominent soft-core gene family in our analysis. A list of all soft-core genes, their predicted functions, and the number of jumbo phage genomes they are present is given in Fig. 2C. Remarkably, six of the seven soft-core genes were associated with nucleic acid metabolism including replication, repair, and nucleotide synthesis, underscoring their significance in jumbo phage evolution. The accessory and unique genomes consisted of 13,205 and 14,062 gene clusters, constituting 48% and 51% of the pan-genome, respectively (Fig. 2B). The absence of core genes and the presence of only seven soft-core genes across all jumbo phages suggest that they form an open pan-genome.

To find the distribution of all soft-core genes among jumbo phages, we categorized all jumbo phages into six genera (each having >10 individual phages), namely *Phikzvirus*, *Machinavirus*, *Schizotequatrovirus*, *Agricanvirus*, *Asteriusvirus*, and *Shapirovirus*, and an “other genera” category with fewer phages (Fig. 2D) and (Table S3). Our observations revealed that only 8.8% (29/331) of the jumbo phages harbored all seven soft-core genes within their genomes. This phenomenon was consistently observed in all jumbo phages belonging to the genus *Schizotequatrovirus* (10/10) and certain jumbo phages from genera such as Unclassified *Tevenvirinae* (4/8), *Ceceduovirus* (1/7), *Eneladuvirus* (5/6), *Pseudotevenvirus* (1/3), *Mimasvirus* (1/2), and unclassified bacterial viruses (7/118). Conversely, 4.8% (16/331) of the jumbo phages lacked any of the seven soft-core genes. This group included *Shapirovirus* (9/10), unclassified *Siphoviridae* (4/8), *Bertelyvirus* (2/2), and unclassified bacterial viruses (1/118), as highlighted in Table S3. Notably, within the genus *Shapirovirus*, all phages, except one, lacked any of the soft-core genes. Notably, 35.6% (118/331) of jumbo phages remained unclassified. Thus, while soft-core genes exhibited differential distribution across all jumbo phages, their distribution patterns remained largely conserved at the genus level.

### Phylogenetic analysis of soft-core genes

**Fig 3:**
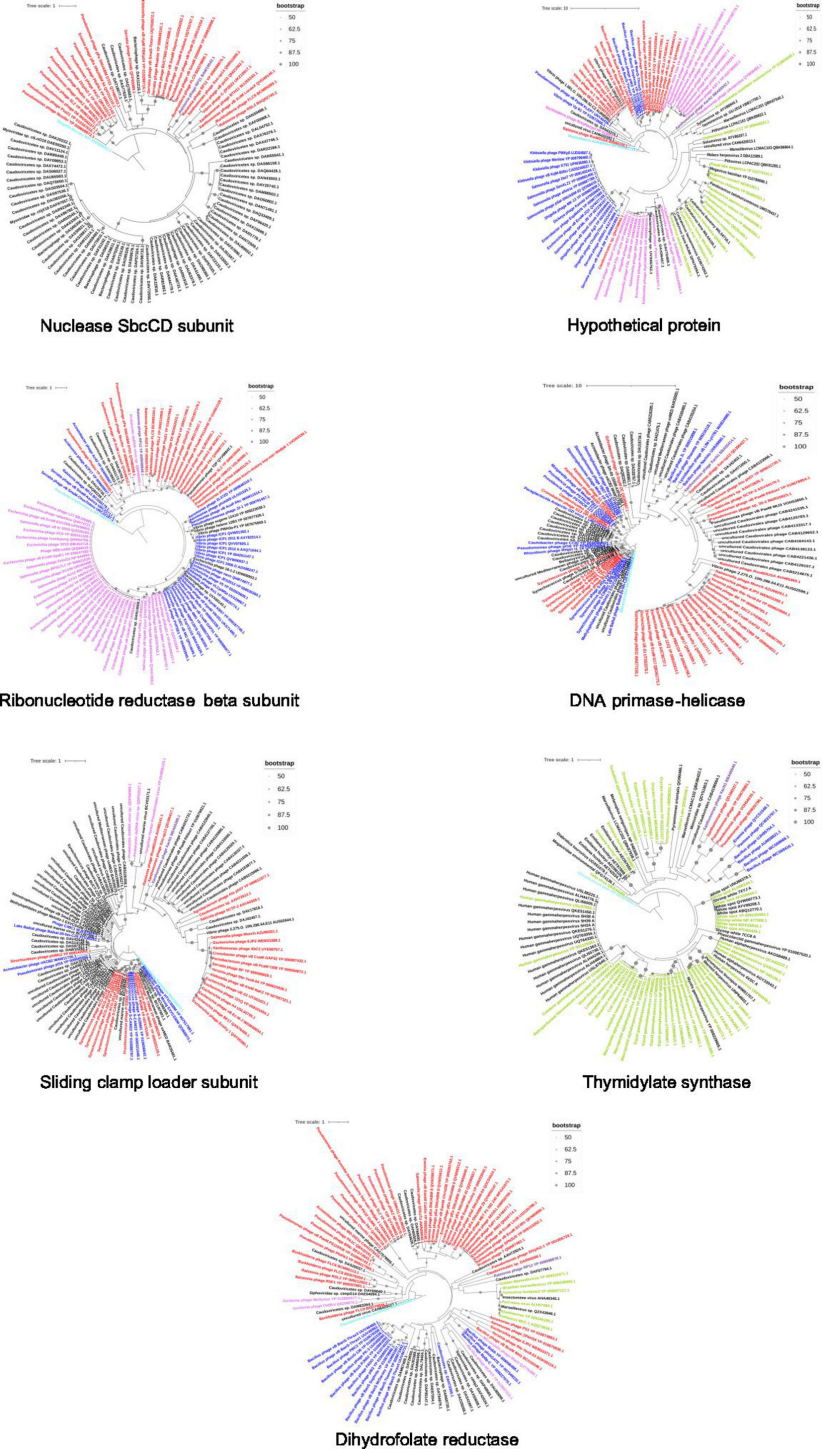
Phylogenetic tree of soft-core genes. Phylogenetic trees of soft-core genes are shown with the names of the genes indicated at the bottom of each tree. In these trees, nodes representing jumbo phages (>180 kb) are highlighted in red. Non-jumbo phages with genome sizes less than 100 kb and between 101-179 kb are shown in pink and blue, respectively. Eukaryotic viruses are indicated in green. Nodes related to the outgroup, query, and MAGs are colored neon-blue, purple, and black, respectively

To elucidate whether the seven soft-core genes are specific to jumbo phages or are also prevalent in other viruses, we conducted a phylogenetic analysis to explore their evolutionary relationships. To accomplish this, we identified the top 100 closest homologs for each of the soft-core genes against the NR database (viruses) using BLASTP-based homology search, aligned them, and further constructed a phylogenetic tree as depicted in Fig. 3A through G.

Our analysis suggested that closest homologs of four of the soft-core genes (D subunit of the SbcCD nuclease (cluster A), ribonucleotide reductase beta subunit (cluster C), DNA primase-helicase (cluster D), and sliding clamp loader subunit (cluster E) show taxonomic distribution across a spectrum of viruses, encompassing jumbo phages, non-jumbo phages, and metagenomic assembled genomes (MAGs), having draft and complete genome assemblies. Given their presence in both jumbo phages and non-jumbo phages, it is evident that these genes are not exclusive to jumbo phages. It is possible that they evolved in a common ancestor preceding the emergence of jumbo phages and played pivotal roles in the phage life cycle.

In contrast, the remaining three soft-core genes, namely hypothetical protein (cluster B), thymidylate synthase (cluster F), and dihydrofolate reductase (cluster G), were not only prevalent in jumbo phages and non-jumbo phages but were also detected in eukaryotic viruses, including giant viruses. This observation suggests potential horizontal gene transfer events across diverse bacteriophages and eukaryotic viruses, as previously reported (41–43) Alternatively, these genes might represent some of the important viral genes that have remained relatively conserved throughout viral evolution due to their pivotal functional roles. Taken together, these observations indicate a polyphyletic nature of the evolution of jumbo phages, reflecting complex evolutionary history and origination via multiple independent ancestors rather than through a common evolutionary ancestor.

### Gene sharing network of jumbo phages

Although our pan-genome analysis revealed the distribution of protein sharing among 331 jumbo phages, grouping and classification could not be achieved based on the pan-genome, and 35.6% (118/331) of jumbo phages remained unclassified. To categorize all 331 jumbo phages into viral clusters, we utilized the vConTACT2 tool (44, 45). This tool computes viral clusters (VCs) based on genome similarities and sharing of orthologous genes, presenting results in nodes and edges. Nodes represent phage genomes, whereas edges quantify the degree of similarity between them, with shorter edges indicating higher shared protein clusters and longer edges signifying lower shared protein clusters. Our analysis resulted in a network comprising 327 nodes of jumbo phages and 4930 edges. Four jumbo phages—*Microcystis* phage MaAM05, *Bacillus* phage SP-15, *Bacillus* phage G, and *Clostridium* phage cst—did not exhibit associations with any other jumbo phages, forming singletons within the jumbo phage gene sharing network (Table S4A).

**Fig 4:**
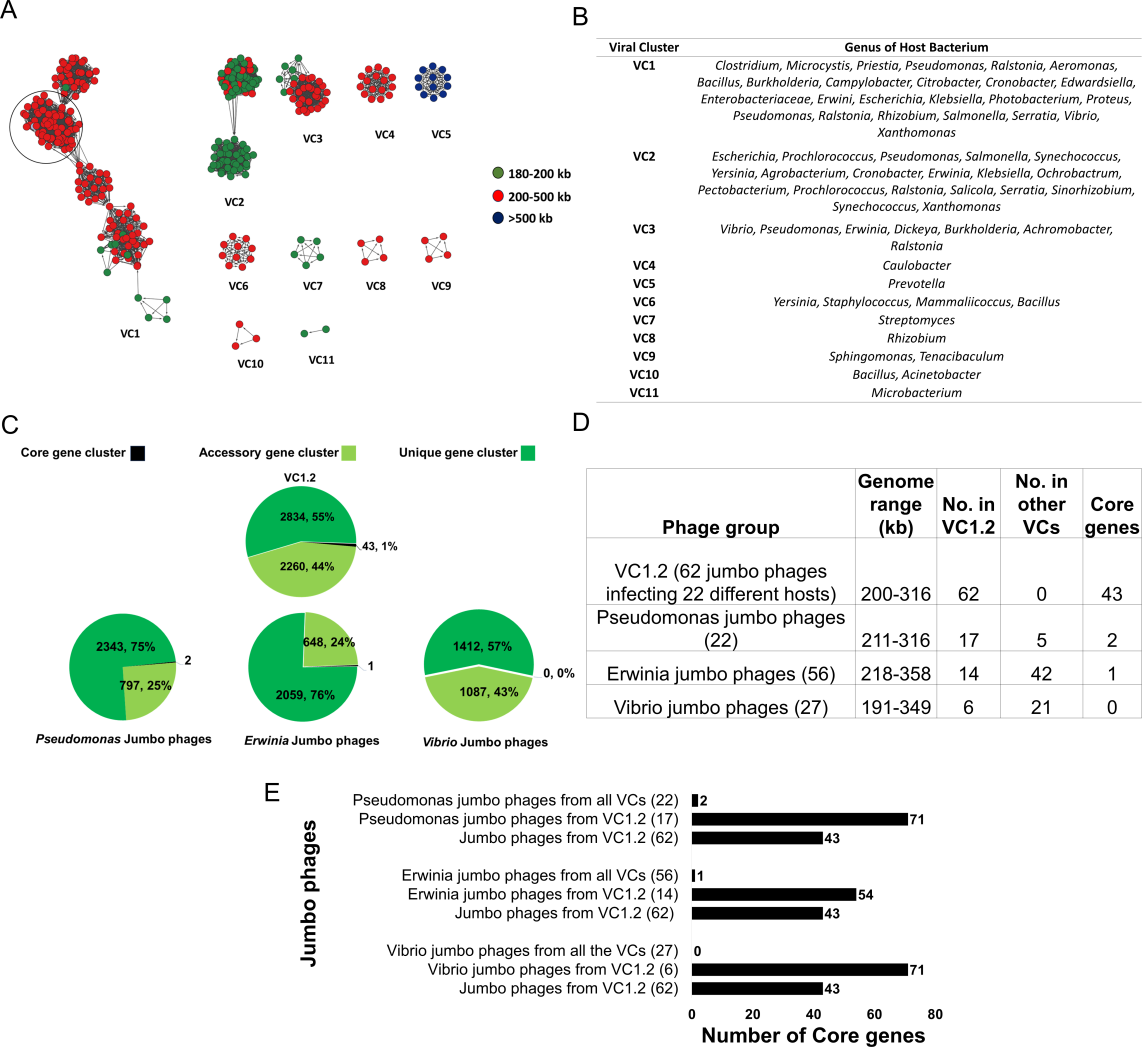
Protein sharing network of jumbo phages with other bacterial viruses. (**A**) A network representation was generated using Cytoscape, where individual phage genomes are depicted as nodes. These nodes are color-coded based on the phage’s genome size distribution, with genomes 180–200 kb (green), genomes 200–500 kb (red), and >500 kb (blue). The connecting lines (edges) between nodes signify the percentage of shared proteins among the phages. This visualization highlights the emergence of 11 major VCs of jumbo phages. (**B**) The figure elaborates on the genus-wide distribution of bacterial hosts of jumbo phages in the corresponding major viral clusters. (**C**) Pie chart depicting the distribution of core, accessory, and unique gene clusters among jumbo phages from VC1.2, as well as those infecting *Pseudomonas*, *Erwinia,* and *Vibrio* hosts distributed across all VCs. (**D**) The table summarizes details of jumbo phages present in VC1.2, as well as *Pseudomonas, Erwinia*, and *Vibrio* jumbo phages listing various information including the number of core genes infecting the respective group. (**E**) Bar graph showing the distribution of core genes across jumbo phages present in VC1.2, *Pseudomonas, Erwinia*, and *Vibrio* jumbo phages.

Based on the analysis, 327 jumbo phages were grouped into 11 major VCs with 130 sub-clusters (Table S4B). Major viral clusters VC1 and VC2 were further divided into five and two minor VCs, respectively (Fig. 4A). This clustering underscores the inherent diversity of jumbo phages and aligns with a previous report that categorized jumbo phages into 11 clusters based on genomic similarity (4). To understand the distribution of jumbo phages as per their genome range, we represented potential jumbo phages with a genome range of 180–200 kb as green nodes, whereas strict jumbo phages (200–500 kb) and megaphages (> 500 kb) were represented as red and blue nodes, respectively. In the viral network, it is evident that potential jumbo phages exhibit extensive gene-sharing networks with strict jumbo phages and are part of the same viral clusters as observed in VC2, VC3, VC7, and VC11. However, megaphages formed a separate cluster (VC5). Thus, it is clear that phages between 180 and 500 kb genome share an overlapping proteome and evolutionary relatedness, consistent with previous suggestions (5).

We next assessed the genus-level distribution of jumbo phages within the VCs. Four major viral clusters (VC4, VC7, VC9, and VC11) contain jumbo phages of the same genera, and seven major viral clusters—VC1, VC2, VC3, VC5, VC6, VC8, and VC10—contain jumbo phages infecting diverse hosts and thus forming multi-genus viral clusters (Fig. 4B; Table S4B). For instance, major cluster VC1 contains 161 jumbo phages infecting 52 different hosts and VC2 contains 87 jumbo phages infecting 37 different hosts. We sought to understand why certain jumbo phages are part of multi-genus viral clusters. To address this, we focused on sub-cluster VC1.2, which comprises 62 jumbo phages with genome sizes ranging from 200 to 317 kb and infecting 22 distinct hosts. VC1.2 presented a unique opportunity to explore the underlying reasons for their clustering despite their varied host ranges. We uncovered the pan-genome of VC1.2 to understand the level of genomic relatedness. A total of 20,404 proteins in VC1.2 were distributed into 5,137 orthologous protein clusters (Table S6A). Among these, 43 orthologous clusters constituted the core genome shared among the 62 diverse jumbo phages within the sub-cluster, whereas 2,260 and 2,834 orthologous protein clusters represented accessory and unique genomes, respectively (Fig. 4C).

We wanted to compare the pan-genome distribution of VC1.2 with specific jumbo phage genera distributed across all viral clusters. For this purpose, we focused on *Pseudomonas*, *Erwinia*, and *Vibrio* jumbo phages to explore their similarities and diversity using pan-genome analysis. Of a total of 22 *Pseudomonas* jumbo phage genomes, a subset of 17 was found in sub-cluster VC1.2, with the remainder distributed across five major VCs. Interestingly, the pan-genome of *Pseudomonas* jumbo phages distributed across all VCs exhibited only two core genes among 3,142 orthologous protein clusters. This starkly contrasts with the core genome composition of sub-cluster VC1.2, which has 43 core orthologous protein clusters. However, the number of core genes increases to 71 if we include only 17 *Pseudomonas* jumbo phages belonging to VC1.2 (Fig. 4D and E). A similar trend was observed when we compared the pan-genomes of *Erwinia* and *Vibrio* jumbo phages spanning multiple clusters with sub-cluster VC1.2. *Erwinia* jumbo phages (*n* = 56), which are distributed in five VCs (VC1.1, VC1.2, VC1.3, VC2.1, and VC3), contain one core orthologous protein clusters, whereas zero core orthologous protein clusters were present in *Vibrio* jumbo phages (*n* = 27) distributed across six VCs (VC1.1, VC1.2, VC1.3, VC1.4, VC1.5, and VC3). These numbers were also significantly less than the 43 core orthologous protein clusters present in the multi-genus viral sub-cluster VC1.2, which included 14 *Erwinia* and 6 *Vibrio* jumbo phages. Contrary to this, if we analyze *Erwinia* and *Vibrio* jumbo phages belonging to VC1.2 only, the number of core genes increases to 54 and 71, respectively (Fig. 4D and E). Overall, these findings underscore the higher degree of genomic conservation and interconnectivity within multi-genus viral clusters, raising the possibility that they originated through a common ancestor, or they are part of broad-host-range phages, or both.

### Analysis of CRISPR-Cas evasion potential of jumbo phages

Bacteriophages employ various strategies to evade CRISPR-Cas immunity, including DNA modification, the deployment of anti-CRISPRs, and mutagenesis of the target sites (46, 47) ⏀KZ-like jumbo phages have been implicated in evading CRISPR-Cas immunity through a unique mechanism, wherein they physically protect their replicating DNA within a specialized phage nucleus (19, 20). This structure is formed by the ChmA protein and positioned by the PhuZ cytoskeletal system (20, 26, 30). These distinctive phages have been classified into a novel family known as Chimalliviridae (29, 30). The Chimalliviridae family represents only a subset of jumbo phages, and the extent to which various other jumbo phages can evade CRISPR-Cas immunity remains unclear. Because genome protection plays an important role in genome evolution, our aim was to analyze the CRISPR-Cas evasion capabilities of jumbo phages by investigating the presence of hallmark genes related to CRISPR-Cas targeting. This includes *chmA* and *phuZ* genes, which encode proteins for phage nucleus formation and localization, respectively (27, 30). Additionally, we also assessed proto-spacers, which are DNA sequences targeted by CRISPR-Cas systems for cleavage (48), and anti-CRISPR proteins (Acrs), which inhibit CRISPR activity by binding to CRISPR-associated proteins (49, 50), as their presence indicates CRISPR-Cas targeting of the phage genome.

**Fig 5:**
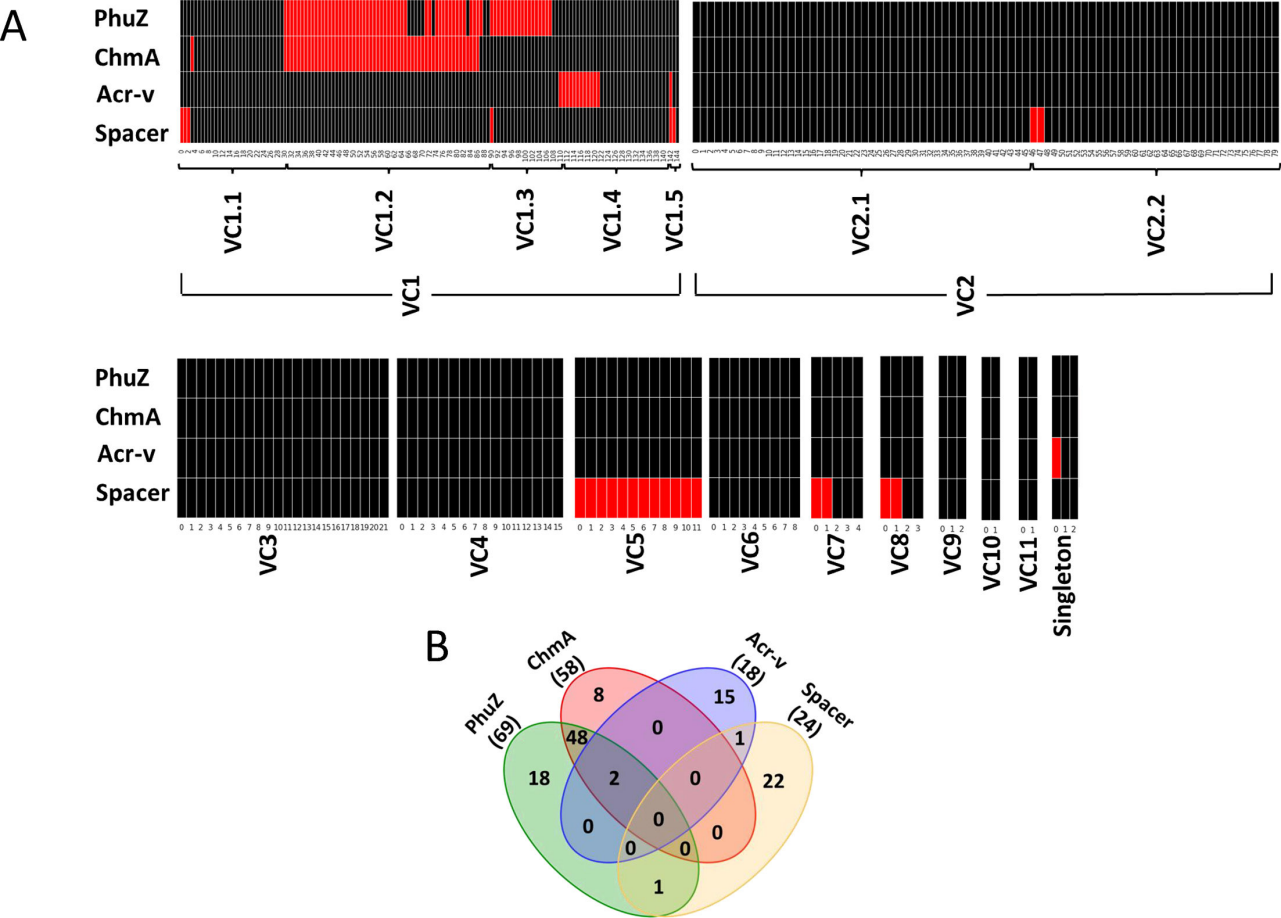
CRISPR targeting analysis of jumbo phages. (**A**) The heatmap illustrates the distribution of *chmA*, *phuZ*, Acr-v, and spacer among jumbo phage genomes, organized by their respective VC. In this visualization, the presence of hallmark genes related to CRISPR-Cas targeting is depicted by red boxes, whereas their absence is denoted by black boxes. (**B**) The Venn diagram provides a visual representation of the number of jumbo phages with *chmA*, *phuZ*, Acr-v, and spacer in relation to each other, offering insights into their patterns of presence and absence across the jumbo phage genomes analyzed.

In our study, 331 jumbo phages have been identified to be associated with 80 host organisms (Table S1). However, among these, only 62 hosts were found to encode CRISPR-Cas defense systems [Table S7A, as determined by using the CRISPR Casdb-taxo tool from the CRISPR Cas ++ database (51)]. As a result, we focused our analysis on the subset of 302 jumbo phages capable of infecting these 62 hosts equipped with CRISPR-Cas systems. Jumbo phages were grouped as per the VCs, and the presence or absence of specific markers is indicated in red or black, respectively. As can be seen in Fig. 5A, *chmA* and *phuZ* were present exclusively in VC1, specifically in the sub-cluster VC1.2. Sub-cluster VC1.3 contains *phuZ*, but not *chmA*. It is likely that an alternative nucleus-forming protein is present in VC1.3 or a highly diverged *chmA* is present, which is below our significant value of detection. Importantly, no spacer sequences or verified Acrs (Acr-v) were detected in jumbo phages of VC1.2 and VC1.3, except for one phage (*Pseudomonas* phage EL) in VC1.3, which has *phuZ* as well as a spacer. This supports previous observations that nucleus-forming jumbo phages effectively evade DNA targeting CRISPR-Cas systems (19, 20) and validates our *in silico* CRISPR-Cas targeting analysis.

Regarding Acrs, we assessed the presence of 135 verified Acrs [as listed in (52)] in 302 jumbo phages. We detected only three unique Acr-v in 18 jumbo phages, which are majorly distributed in VC1.4 and VC1.5 (Fig. 5A; Table S7B). In the case of spacers, we identified a total of 57 spacers within the CRISPR arrays targeting 24 of the 302 jumbo phages examined (Fig. 5A and B; Table S7D and E). Some jumbo phages were targeted by multiple spacers. Spacers were detected not only in all jumbo phages in VC5, which are *Prevotella*-infecting megaphages, but also in some jumbo phages distributed across VCs. The presence of Acr-v and spacer match indicates that some jumbo phages are clearly targeted by the CRISPR-Cas system. However, our study identified 187 of 302 jumbo phages lacking *phuZ*, *chmA*, Acr-v, or spacers. These phages constituted a substantial 61% of phages, distributed across all VCs except VC5, and infecting CRISPR-Cas containing the host. These jumbo phages appeared to possess yet-to-be-identified mechanisms for evading CRISPR-Cas targeting. Taken together, our results suggest that a majority of jumbo phages possess mechanisms to circumvent the CRISPR-Cas system, employing both phage nucleus-dependent and phage nucleus-independent strategies, potentially involving novel mechanisms.

## DISCUSSION

Jumbo phages are a novel group of bacteriophages with large genomes; however, their evolutionary trajectory, genetic makeup, and biological characteristics remain enigmatic. In this study, we attempted to understand the pangenomic diversity, evolutionary relatedness, and CRISPR-Cas evading potential of jumbo phages by conducting a comprehensive genomic analysis involving 331 jumbo phages having complete genomes and capable of infecting 80 bacterial genera. Homology-based characterization of their proteins revealed a complex repertoire of functions, including an abundance of genes associated with DNA replication, recombination, repair, as well as metabolic and signaling functions. Given their prolonged replication period within their host, these proteins are likely deployed for extended DNA replication, DNA repair, and host take-over processes. Our study leveraged two distinct bioinformatics tools to predict the functions of nearly 27% of the jumbo phage proteins. Nevertheless, the functions for the majority of jumbo phage genes remain unknown due to the lack of sequence homology or functional domains comparable with known proteins. Importantly, some of the uncharacterized genes exhibit high conservation, suggesting important roles in the jumbo phage life cycle. These repertoires of uncharacterized jumbo phage genes constitutes a genomic dark matter, and future investigations involving combinations of structural analyses, functional genomics, and meta-proteomics might reveal the function of these enigmatic jumbo phage genes.

Our pan-genome analysis points out that jumbo phages form an open pan-genome. Moreover, we could not detect core genes that are conserved across all jumbo phages. This is in contrast to observations made by (5), which suggested that several genes including terminase large subunit and chromosome-end processing complex SbcCD are universally conserved across jumbo phages (5). The difference comes because of the methodological approach. In our pan-genome analysis, sequence conservation with a threshold of 25% identification and 0.95 of similarity was used to classify genes. A similar cutoff is commonly used in many pan-genome-based studies (53–55). However, Iyer et al. 2020 used functional conservation to understand the distribution of genes. In this way, they were able to identify several conserved genes of jumbo phages, although they do not have sequence similarity (5). Irrespective of the approach, our analysis identified chromosome-end processing complex SbcCD D-subunit as the top-most conserved soft-core gene (cluster 1), and the same gene was also identified in their approach (5). Pan-genome analysis through sequence conservation revealed additional genes that were not previously detected. This includes other soft-core genes identified in our study namely, a hypothetical uncharacterized protein (cluster 2), ribonucleotide reductase beta-subunit (cluster 3), DNA primase-helicase (cluster 4), sliding clamp loader subunit (cluster 5), thymidylate synthase (cluster 6), and dihydrofolate reductase (cluster 7). Although jumbo phage genomes display extreme sequence divergence, conservation of the soft-core genes suggests that they play important roles in their life cycle, especially in the process of DNA replication and repair and nucleotide synthesis, as predicted by their function. Furthermore, the distribution pattern of soft-core genes is largely conserved at the genera level. A similar genera-level conservation was recently observed in the Chimalliviridae family, which possesses a core set of 72 genes (29). These genes are notably absent in other jumbo phages. Comparative genome analysis has led to the identification of critical proteins that play a significant role in phage nucleus formation within the Chimalliviridae family (29). Our analysis provides information on relatively highly conserved genes across all jumbo phages, as well as highly conserved genes within each family. Thus, future experimental investigations focusing on these genes can lead to the elucidation of critical genes important for the life cycle of jumbo phages in various families.

Jumbo phages are polyphyletic, indicating their lack of a common ancestor (5, 12). Current models propose multiple independent origins of jumbo phages from smaller phages, as they are found in the Myoviruses and Siphoviruses families (4, 5). Interestingly, a phylogenetic analysis of soft-core genes reveals two major trends: (i) all soft-core genes are present in non-jumbo phages, suggesting that they are not unique to jumbo phages and may serve essential functions in other phages as well. (ii) Some of the soft-core genes are conserved in giant eukaryotic viruses, indicating possible horizontal gene transfer events or general conservation among viruses. Such intriguing gene conservations between bacteriophages and giant eukaryotic viruses have been observed before (41, 42).

Our attempt to categorize jumbo phages through gene-sharing networks revealed 11 major viral clusters. Within these clusters, phages spanning a genome size range of 180 kb to 500 kb clustered together due to overlapping gene-sharing networks and evolutionary relatedness in contrast to megaphages with genome sizes exceeding 500 kb, which formed a separate viral cluster. These observations support previous findings (5) and suggest that phages with genome sizes ranging 180–200 kb can be considered potential jumbo phages due to their shared genomic landscapes with jumbo phages with genome sizes between 200 and 500 kb. An intriguing observation in the jumbo phage viral network is the prevalence of multi-genus viral clusters, where jumbo phages infect diverse hosts. Although some clusters exclusively comprise jumbo phages infecting the same host, host specificity does not necessarily dictate membership in most clusters. It is conceivable that multi-genus viral clusters are enriched with jumbo phages exhibiting broad host ranges. Recently, highly broad-spectrum phages have been identified to infect diverse hosts based on the receptors encoded by conjugative plasmids (56, 57). Although it remains uncertain whether jumbo phages belonging to multi-genus viral clusters are part of this phenomenon, their clustering and grouping suggest origin from common ancestors and evolutionary relatedness. Further investigation into ecological roles and phage tropism of multi-genus viral clusters is required to elucidate their infection dynamics and ecological significance.

Due to the presence of large genomes, jumbo phages appear to have evolved specialized systems to protect their genetic material. Chimalliviridae family of phages employ phage nucleus compartments to protect the replicating DNA from immune systems like DNA-targeting Cas enzymes (19, 20, 26). Additionally, the presence of spacer sequences that match phage DNA and anti-CRISPR proteins in the phage genome are indications of CRISPR-Cas targeting (48, 58, 59). In our study, we looked at the CRISPR-Cas targeting potential of jumbo phages, which were grouped based on their viral clusters. Jumbo phages equipped with ChmA, PhuZ, or both are generally not targeted by the host CRISPR-Cas, as evidenced by the lack of spacers and verified anti-CRISPRs in their genomes. This aligns with the prevailing model, indicating that jumbo phages forming protective phage nuclei are not targeted by the host CRISPR-Cas systems (27) and serves as a validation of the *in silico* CRISPR-Cas analysis. However, a notable observation is that a substantial portion of jumbo phages, that is, nearly 61% of jumbo phages infecting CRISPR-Cas containing host, appear to evade the CRISPR-Cas targeting while simultaneously lacking the hallmark genes for phage nucleus formation, anti-CRISPRs, or spacer sequences. These phages are distributed across viral clusters. This intriguing phenomenon suggests that they must be employing hitherto unidentified mechanisms to circumvent CRISPR-Cas immunity. It is conceivable that certain jumbo phages employ genome modifications, similar to known mechanisms (60–62), to safeguard their genetic material from CRISPR-Cas targeting. It is equally possible they use novel DNA modifications or as-yet-undiscovered mechanisms to protect their genome. It is essential to conduct additional experimental investigations beyond Chimalliviridae family of phages to further our knowledge of the enigmatic interaction of jumbo phage genome and host CRISPR-Cas systems. Such investigations will unveil the diversity of strategies employed by phages to overcome the CRISPR-Cas system.

## MATERIALS AND METHODS

### Data collection

Data related to the whole-genome sequences of Jumbo-phage were collected from the NCBI Virus database (https://www.ncbi.nlm.nih.gov/labs/virus/vssi/#/) as of 30 August 2021. Data filtration of 538 jumbo phages was performed to remove redundant copies. Upon filtration, we obtained a list of 331 jumbo phages with more than 180 kb genome size, for which the complete genome sequence was available (RefSeq Assembly). Our data set includes 62 phages with 180 to 200 kb genomes (potential jumbo phages), 257 phages with 200–500 kb genomes (strict jumbo phages), and 12 phages with genomes > 500 kb (megaphages). In our study, all these phages (*n* = 331) are taken into consideration as jumbo phages. The NCBI Batch Entrez nucleotide database was used to download all the genome sequences (https://www.ncbi.nlm.nih.gov/sites/batchentrez). Accession numbers and related information regarding the genomes are provided in Table S1.

### Genome annotation pipelines and databases

Genomes were annotated in batches using Rapid Annotation using the Subsystem Technology Pipeline (RASTtk, https://rast.nmpdr.org/rast.cgi) (63). Using hmmscan module from the HMMER suite (http://hmmer.org), all jumbo phage genomes were scanned against the Pfam-A database (https://www.ebi.ac.uk/interpro/) with an E-value cutoff of 1e^−5^ and further parsed using hmmscan-parser.sh to identify confirmatory non-overlapping domains of known functions and motifs within all genes.

### Pan-genome and phylogenetic analysis

Proteinortho v6.0.12 (40) pipeline was used for the pan-genome study with an E-value cutoff of 1E^−05^ and other default settings. It identifies orthologous protein sequences using the reciprocal blast hit (RBH) strategy by utilizing Diamond v0.836.98 and BLAST 2.8.1+. BLASTP search was performed against the NR-Database (Viruses) to identify viral homologs, and the top 100 hits with significant E-values (1e^−05^) were used for phylogenetic analysis. We used MUSCLE v3.8.1551 and MEGAX v11.0.13 for alignment and phylogeny, respectively (64). The RAxML v8.2.12 program was used to generate maximum likelihood phylogenies of aligned genes using PROTGAMMAI (mtREV24-G-I-F) as an amino acid substitution model (−m) with a bootstrap value of 100. An online iTOL platform (65) was used to visualize the phylogenetic trees.

### Phage genome comparisons and visualization

vConTACT (https://bitbucket.org/MAVERICLab/vcontact2) MCL clustering (PC method-MCL, VC method- ClusterONE, PC-inflation and VC-inflation—1.8, Reference database—Prokaryotic Viral RefSeq version 201 with ICTV-only taxonomy) was used to predict protein-protein phage genome comparisons (44, 45). The phage genome comparison network was visualized using Cytoscape (66) (http://www.cytoscape.org/), and all duplicated edges were removed.

### Prediction of CRISPR-Cas systems in bacterial hosts

In this study, 80 different hosts were assigned for 331 jumbo phages. In order to identify which of these hosts contain CRISPR-Cas systems in their genome, we employed the CRISPR Casdb-taxo tool from the CRISPR Cas ++ database (https://crisprcas.i2bc.paris-saclay.fr/MainDb/StrainList) (51). The database provides information regarding the the presence or absence of CRISPR-Cas system in the respective host.

### CRISPR-spacer analysis of jumbo phages

For CRISPR spacer analysis, we utilized the web server for the CRISPR Spacer Database and Exploration Tool (http://crispr.genome.ulaval.ca/). This database comprises over 11 million spacers (48), allowing it to predict spacers that match the input phage sequence, providing host information and their genomic positions. In our study, we considered a maximum of two mismatches between host spacers and phage protospacers for positive hits as previously described in (67). Only spacers that matched the appropriate phage were considered positive hits. For instance, a Pseudomonas jumbo phage with a spacer hit in a Pseudomonas host is considered a positive hit, whereas in a non-Pseudomonas host, it is not considered a positive hit in our analysis.

### Acr analysis

To identify anti-CRISPRs within the jumbo phage genomes, we utilized the data set presented in (52). In brief, we retrieved information corresponding to a total of 135 unique verified anti-CRISPRs listed in (52), which included set A verified, set B verified, and set A literature. Putative Acrs mentioned in (52) were excluded from consideration. The list of Acrs analyzed in this study is provided in S7C. We conducted a Blastp search of 118,833 protein-encoding ORFs from 331 jumbo phages against the 135 Acrs to detect hits. Hits with an e-value of 1e^−5^ and identity exceeding 30% were considered positive hits.

## Data Availability

Data used in study were obtained from open-source databases such as NCBI. All data generated in this study are provided as supplementary information.

## SUPPLEMENTAL MATERIAL

Table S1 spectrum.04200-23-s0001.xlsx General information of jumbo phages (S1A).

Table S2A-Set 1 spectrum.04200-23-s0002.xlsx RASTtk-based annotated protein sequences of jumbo phage (S2A)-Set 1.

Table S2A-Set 2 spectrum.04200-23-s0003.xlsx RASTtk-based annotated protein sequences of jumbo phage (S2A)-Set 2.

Table S2B spectrum.04200-23-s0004.xlsx Pfam-mapping based annotation (S2B).

Table S3 spectrum.04200-23-s0005.xlsx Heatmap of soft-core gene distribution in jumbo phage genomes.

Table S4 spectrum.04200-23-s0006.xlsx Distribution of viral clusters and viral sub-clusters in jumbo phages (S4A).

Table S5 spectrum.04200-23-s0007.xlsx Distribution of jumbo phages and their genome size based on the assigned viral clusters.

Table S6 spectrum.04200-23-s0008.xlsx Pan-genome data of jumbo phages from VC1.2 (S6A), Pseudomonas (S6B), Erwinia (S6C), and Vibrio (S6D).

Table S7 spectrum.04200-23-s0009.xlsx Distribution of Acr, spacer across jumbo phages infecting CRISPR-Cas containing hosts.

ASM does not own the copyrights to Supplemental Material that may be linked to, or accessed through, an article. The authors have granted ASM a non-exclusive, world-wide license to publish the Supplemental Material files. Please contact the corresponding author directly for reuse.
